# Comparative analysis of farmer practices and high yield experiments: Farmers could get more maize yield from maize-soybean relay intercropping through high density cultivation of maize

**DOI:** 10.3389/fpls.2022.1031024

**Published:** 2022-11-15

**Authors:** Guopeng Chen, Yongfu Ren, Atta Mohi Ud Din, Hina Gul, Hanlin Chen, Bing Liang, Tian Pu, Xin Sun, Taiwen Yong, Weiguo Liu, Jiang Liu, Junbo Du, Feng Yang, Yushan Wu, Xiaochun Wang, Wenyu Yang

**Affiliations:** ^1^ College of Agronomy, Sichuan Agricultural University, Chengdu, China; ^2^ Sichuan Engineering Research Center for Crop Strip Intercropping System, Key Laboratory of Crop Ecophysiology and Farming System in Southwest China (Ministry of Agriculture), Chengdu, China; ^3^ Agriculture Technology Extension Station, Liangzhou County Bureau of Agriculture and Rural Affairs, Wuwei, China; ^4^ Key Laboratory of Crop Physiology Ecology and Production Management, Ministry of Agriculture, Nanjing Agricultural University, Nanjing, China; ^5^ National Research Center of Intercropping, The Islamia University of Bahawalpur, Bahawalpur, Pakistan; ^6^ National Center of Industrial Biotechnology, Pir Mehr Ali Shah Arid Agriculture University Rawalpindi, Shamsabad, Pakistan; ^7^ Agriculture Technology Extension Station, Pingchang County Bureau of Agriculture and Rural Affairs, Bazhong, China

**Keywords:** intercropping, leaf angle, leaf area index, photosynthetic rate, yield

## Abstract

Intercropping is a high-yield, resource-efficient planting method. There is a large gap between actual yield and potential yield at farmer’s field. Their actual yield of intercropped maize remains unclear under low solar radiation-area, whether this yield can be improved, and if so, what are the underlying mechanism for increasing yield? In the present study, we collected the field management and yield data of intercropping maize by conducting a survey comprising 300 farmer households in 2016-2017. Subsequently, based on surveyed data, we designed an experiment including a high density planting (Dense cultivation and high N fertilization with plough tillage; DC) and normal farmer practice (Common cultivation; CC) to analyze the yield, canopy structure, light interception, photosynthetic parameters, and photosynthetic productivity. Most farmers preferred rotary tillage with a low planting density and N fertilization. Survey data showed that farmer yield ranged between 4-6 Mg ha^-1^, with highest yield recorded at 10-12 Mg ha^-1^, suggesting a possibility for yield improvement by improved cropping practices. Results from high density experiment showed that the two-years average yield for DC was 28.8% higher than the CC. Compared to CC, the lower angle between stem and leaf (LA) and higher leaf area index (LAI) in DC resulted in higher light interception in middle canopy and increased the photosynthetic productivity under DC. Moreover, in upper and lower canopies, the average activity of phosphoenolpyruvate (PEP) carboxylase was 70% higher in DC than CC. Briefly, increase in LAI and high Pn improved both light interception and photosynthetic productivity, thereby mediating an increase in the maize yield. Overall, these results indicated that farmer’s yields on average can be increased by 2.1 Mg ha^-1^ by increasing planting density and N fertilization, under plough tillage.

## Introduction

Ever-increasing global population is a continuous challenge, especially for the densely populated countries like China, causing food security problems (Gandhi and Zhou, 2014). One of the key solutions of this problem is to improve the existing crop yield from cultivated lands. Multiple cropping systems like cereal-legume intercropping have been proven to play important role in improving land utilization as compared to mono-cropping system (Li et al., 2016). Therefore, these methods have been widely adopted worldwide in countries like China, America, India, and Africa to increase the crop productivity (Yang et al., 2015). In China, half of the total grain yield is produced through multiple cropping systems and intercropping is practiced on more than 2.8 × 10^7^ ha of the arable land. Traditionally, Chinese farmers have intercropped soybean with wheat, maize, millet, cotton, etc. (Knörzer et al., 2009; Li et al., 2013) but the maize-soybean intercropping is considered the most productive in terms of resource use efficiency and land equivalent ratios. The success of cereal-legume intercropping system profoundly depends on the temporal and spatial complementarity of resource utilization (Xue et al., 2016). Therefore, several studies have been carried out on the critical aspects of intercropping such as varietal breeding and screening, planting pattern (Yang et al., 2015), lodging resistance (Luo et al., 2015), fertilizer management (Yong et al., 2014), water use efficiency and water distribution (Rahman et al., 2017), relative crowding coefficient, competitive ratio, actual yield loss, intercropping advantage indices, growth improvement and light irradiance (Yang et al., 2014). Such studies helped to understand the scientific basis to improve the intercropping systems, however, the knowledge about the actual intercropping practices performed by the farmers is still limited. Therefore, study about the common intercropping practices in farmer’s field could help the researchers to address the yield disparity within farmers which will bring uniformity in the productivity of intercropping systems in the country.

Maize-soybean strip intercropping contains two major systems including traditional strip intercropping and relay-strip intercropping. In maize-soybean relay strip intercropping systems (MSR), the narrow-wide planting pattern is adopted and maize is usually sown either at the end of march or at the beginning of April and harvested in July-August (Yang et al., 2014). Later on, soybean is sown between the wide spaces of maize strips at the beginning of June and harvested in late October (Yang et al., 2014). Therefore, relay intercropping help to grow both crop species during one season, in areas like Sichuan where the growing season is too short for the double cropping (Yang et al., 2015). In recent years, maize-soybean relay strip intercropping system has been popularized in the Southwestern China (Yan et al., 2010) and provided considerable economic and social benefits for small-land hold farmers. Importantly, the southwest China is one of the most densely populated agricultural regions where farmers possess relatively small pieces of cultivated area (some plots less than 500 m^2^ per farmer), thus farmers adopt different cultivation patterns and practices (Chen et al., 2015; Yan et al., 2018; Zhou et al., 2019a). This phenomenon has generated a wide variation in methods used for fertilization, tillage, and varietal choosing (Liu et al., 2021a). Previously, Gou et al., 2017 evaluated yield potential under the intercropping system in Northwest China under abundance solar radiation, more than 6000 MJ m^-2^ per year. They found that the potential yield of intercropped maize was 12.0 t ha^-1^, with an actual yield of 10.1 t ha^-1^ in farmer’s field. Notably, the maize yield increased after input of N- and P-fertilizers, reaching 17.1 t ha^-1^ (Li et al., 2001). The yield increase was largely attribute to the complementarity effect, nutrient input, choosing compact cultivars, and adequate irrigation (Gou et al., 2017; Chen et al., 2019; Li et al., 2020). However, in southwest China which have comparatively lower solar radiation, little is known about the actual yield of maize farmers adopting the relay intercropping system.

In present study, we hypothesized that maize yield under the intercropping system in Southwest China can be improved by adjusting field management and increase in the light interception as well as photosynthetic productivity. Therefore, we collected and analyzed field management and yield data from 300 farmers in Sichuan province over two-years. Subsequently, we designed a high yield experiment for two years, to analyze canopy structure, light interception, photosynthetic parameters, photosynthetic productivity, and yield. The findings of this study provide new insights into the common intercropping practices by the farmers, which could help the future studies to propose a uniform intercropping system in terms of yield and productivity.

## Materials and methods

### Assessment of commonly used farmer practice

We selected and visited 300 farmers for survey in Sichuan province between 2016 and 2017 to assess the commonly used farmer practice for MSR in the Sichuan province. Three counties were randomly selected from Sichuan province. For each county, 10 villages were randomly selected, with each village providing 10 households. All the surveyed farmers were involved in MSR. Data collected from these farmer fields included maize grain yield from intercropped fields, planting density, tillage methods, and N fertilization. For more details about the survey data, please see the Supplementary-Survey data.

### Site and experimental design

**Figure 1:**
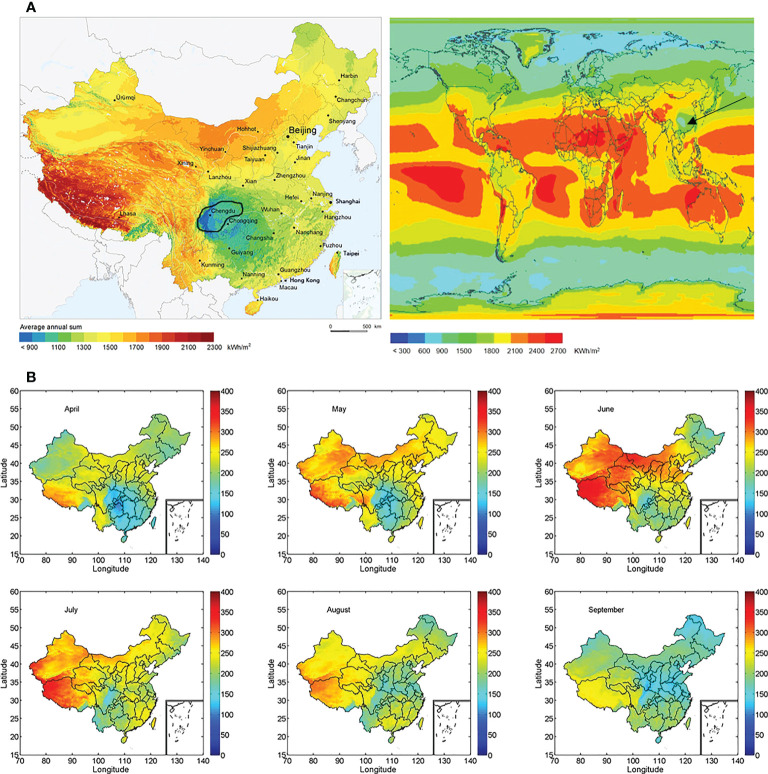
Solar radiation in various regions of China (2021 Copyright Sun Reign Ltd). **(A)**: The black circle indicates Sichuan province. Southwest China (includes the Sichuan, Yunnan, Guizhou provinces, and Chongqing city) are lower solar radiation than surrounding countries. **(B)**: Solar radiation during the maize growth period, the unit of solar radiation is W m^-2^.

Maize (*Zea mays* L.) variety Zhongyu-3 (with a small angle between stem and leaf, and an average of 19 leaves per plant, resistant to ear rot) and soybean (*Glycine max* L.), variety Nandou-12 (shade-tolerant soybean) were used in the present study. The two varieties occupy the largest local planting area under maize and soybean cultivation. Field experiments were carried out at Modern Agriculture Expert Compound Renshou County, Sichuan Province, China (29°60′ N, 104°00′ E). The study site had an average annual air temperature of 17.4 °C, precipitation of 1009.4 mm, sunshine of 1196.6 h, and lower solar radiation of 3580 MJ m^-2^ (**Figure 1**) (Gajipra, 2015; Zhou et al., 2019b). Details on solar radiation of maize at key stages, namely V6, V14, R2, are shown in **Figure 1B** (Tang et al., 2015).

**Figure 2:**
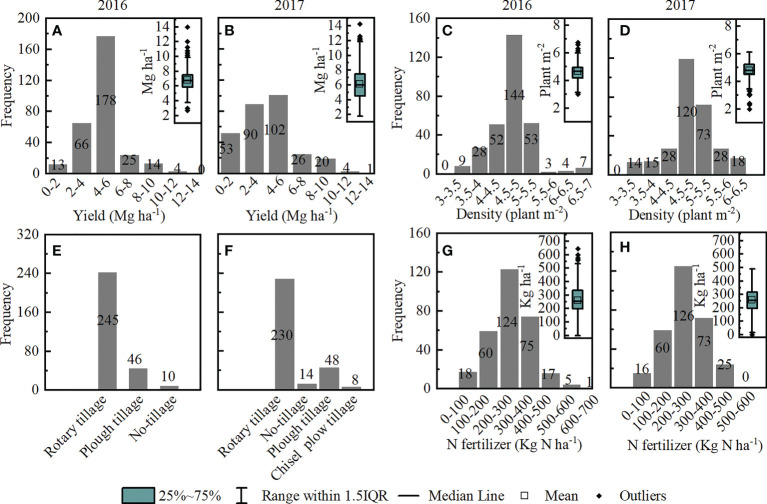
Statistics for yield, planting density, tillage methods, and nitrogen (N) fertilizer survey data of relay intercropping maize from farmers. **(A, B)**: grain yield in 2016-2017. **(C, D)**: the frequency distribution histogram and boxplot of planting density in 2016 and 2017. **(E, F)**: the frequency distribution histogram of tillage methods in 2016 and 2017. **(G, H)**: the histogram and boxplot of N fertilizer in 2016 and 2017; n = 300.

Based on the information obtained from the farmer field survey, we designed a field experiment to assess the response of maize yield components to high density planting (Dense cultivation; DC) as compared to normal farmer practice (Common cultivation; CC). In addition, we adopted plough tillage for DC, and added more nitrogen to compensate the competition within maize plants. The CC was designed on the basis of highest frequency from surveyed data (**Figures 2C-H**) and the intercropped maize was planted with the density of five plants m^-2^ and N fertilizer applied at a rate 240 kg N ha^-1^ under rotary tillage (**Figure 2**). DC was designed with high density approach in which intercropped maize was planted at a density of 6.75 plants m^-2^ (the highest density from surveyed data) and N fertilizer applied at a rate of 270 kg N ha^-1^, under plough tillage. The experiments were conducted in a randomized block design, with three replicate blocks and a total of six plots (2 treatments × 3 blocks). Each plot had an area 267 m^2^ (6 m × 44.5 m). Importantly, both CC and DC have same configuration of MSR, i.e., 2M2S (two-rows of soybean were relay-intercropped with two rows of maize after 60 ± 10 days of maize sowing) in which the strip of maize and soybean each had 40 cm width, with 60 cm of space between the strips of maize and soybean (**Figure S1**). The distance of the plant to plant in CC and DC were 20 cm and 15 cm, respectively. Fertilizer, superphosphate, was applied at a rate of 600 kg ha^-1^ (containing 12% P_2_O_5_), and 150 kg ha^-1^ of potassium chloride (containing 60% KCl) for maize in CC and DC. Maize was sown on April 9, 2018, and April 5, 2019, while soybean was sown on June 17 of each year. Manual weeding was performed as per requirement under the rainfed agriculture. Maize harvesting was done on August 5, 2018, and August 9, 2019. Soybean harvesting was done on October 26, 2018, and October 28, 2019. Soybean was planted with the density of 12 plants m^-2^; N fertilizer was applied at a rate of 30 kg N ha^-1^, 30 kg ha^-1^ of potassium chloride (containing 60% KCl), and 30 kg ha^-1^ of superphosphate (containing 12% P_2_O_5_).

### Analysis of plant morphology

LAI (leaf area index), which refers to the leaf area of the unit land area, was calculated using the ratio of leaf area to the maize and soybean planting areas (Liu et al., 2018b). Total LAI at V6 (sixth leaf), V14 (fourteen leaf), R1 (silking), R2 (blister), and R6 (maturity) stages was measured from five randomly selected plants of intercropped maize. Furthermore, the upper (above the three-ear leaves), middle (three-ear leaves), and lower (below the three-ear leaves) canopies LAI were calculated at R1 stage, respectively. Similarly, the other morphological parameters including plant height, ear height, stem diameter, leaf angle (LA, the angle between leaf and stem), and leaf orientation value (LOV) were also measured from five randomly selected plants at R1 stage. Ear height was the distance from the ground to the uppermost ear bearing node. Leaf area of individual leaves was calculated using the following formula according to a method by (Gao et al., 2016).


Leaf area = length x width x 0.75

A protractor was used to measure the upper canopy LA (the average LA of above three-ear leaves); the middle canopy LA (the average LA of three-ear leaves); the lower canopy LA (the average LA of three-ear leaves).

LOV was calculated using the following formula, as previously described (Pepper et al., 1977; Lu et al., 2018):


$$\text{LOV}=\sum_{i=1}^{n}\frac{\left(90-\text{θ}i\right)\left(\frac{Lf}{L}\right)}{n}$$

Where θ*_i_* is the angle between stem and leaf, L denotes leaf length, Lf represents the spatial distance between the leaf collar and leaf tip, whereas n indicates the number of measured leaves. For instance, there are three leaves in middle canopy, middle canopy LOV = (90 – θ_1_)(Lf_1_/L_1_)/3 + (90 – θ_2_)(Lf_2_/L_2_)/3 + (90 – θ_3_)(Lf_3_/L_3_)/3. LOV of the upper and lower canopies were calculated using a similar approach for the middle canopy.

### Determination of light distribution, transmittance, and light interception rate

The measurements were taken on a sunny and cloudless day, between 10:00 AM and 12:00 PM. PAR was measured using a 1-m line quantum sensor (LI COR Inc., Lincoln, NE, USA) and an LI-1400 data logger. Measurements in the canopy were performed at a 30-cm and 20-cm intervals in the vertical and horizontal direction, respectively, at R1 stage in 2018 and 2019 (**Figure S1**). Light transmittances in respective canopies were calculated as follows: upper canopy = I_u_/I_t_ ×100%; middle canopy = I_m_/I_t_ ×100%; lower canopy = I_l_/I_t_ ×100%. On the other hand, light interception rates in respective canopies were calculated as follows: upper canopy = (1-I_u_/I_t_)×100%; middle canopy = (1-I_m_/I_u_)×100%; lower canopy = (1-I_l_/I_m_)×100%. I_t_ is PAR of the top canopy; I_u_, Im, and I_l_ denote PAR of the upper, middle, and lower canopies, respectively (Liu et al., 2018a) (**Figure S1**).

### Analysis of key enzyme activities involved in photosynthesis

Five plants in each plot were randomly selected at the R1 stage, and the activities of Rubisco and PEP carboxylase enzymes was assayed in the upper canopy (fourth leaf above the ear leaf), middle canopy (ear leaf), and lower canopy (fourth leaf below the ear leaf). All leaf samples were immersed in liquid nitrogen and immediately stored at -80 °C for measuring the enzyme activities. Then, we extracted crude enzyme, and measured Rubisco and PEP carboxylase activities according to the previously published methods (Wang et al., 2008; Sui et al., 2017), with slight modifications. 100 mg leaf sample was ground with extraction buffer. Then were centrifuged at 12,000 ×g at 4 °C for 15 minutes. Supernatants were used as crude extract for total activity assays. Activation was performed in a 100 μl mixture solution at 28 °C for 15 minutes. Initial Rubisco activity was determined. The change in the absorbance of NADH was measured at 340 nm within one min. PEP carboxylase activity was measured spectrophotometrically at 340 nm by coupling the PEP carboxylase reaction to the malate dehydrogenase (MDH) reaction, using a buffer with 50 mM bicine (pH 8.2), 2 mM DTT, 5 mM MgCl_2_, 1 mM NaHCO_3_, 1 mM Na_4_EDTA, 0.25 mM glucose-6-phosphate, 0.15 mM NADH, 2 units MDH and 2 mM PEP and enzyme extract. The reaction was initiated by the addition of PEP.

### Determination of the photosynthetic rate and productivity

Photosynthetic activity was measured on a clear and cloudless day, between 10:00 AM and 12:00 PM, at R1 stage. Five plants in each plot were randomly selected and Pn of the upper canopy (fourth leaf above the ear leaf), middle canopy (ear leaf), and lower canopy (fourth leaf below the ear leaf) were determined using LI-6400-XT photosynthetic apparatus (Lincoln, USA). The tests were performed under the following conditions: leaf chamber temperature was set at 25 °C, PAR of 1000 µmol m^-2^ s^-1^, and a CO_2_ concentration maintained at 400 µmol mol^-1^. Photosynthetic productivity was calculated using the Baig formula (Baig et al., 1998) as follows:


Photosynthetic productivity=Pn x LAI.

### Analysis of yield and yield components

An area of 30 m^2^ was selected and effective ear at maturity counted. Twenty ears were selected to determine grain number per ear, and 1000-grain weight (1000-GW), with the yield recorded as follows:


Yield=effective ear x grain number per ear x 1000-GW

(Chen et al., 2019).

### Statistical analysis

Statistical analyses were performed using SPSS software (SPSS 22, SPSS Inc., USA), and difference among groups was determined using one-way analysis of variance (ANOVA) followed by the least significant difference (LSD) multiple-range test. Data followed by *P* < 0.05 was considered statistically significant. Correlation analysis was performed using the Pearson correlation coefficient test, while figures were generated using Origin Pro (version 2019, Origin Lab).

## Results

### Yield, planting density, tillage methods, and N fertilizer survey data

Results from the survey, comprising about 300 farmer’s households showed that most of the intercropping grain yields were 4-6 Mg ha^-1^ in two years. Notably, in 2016 and 2017, 59.3% and 34.5%, respectively, of the surveyed fields had a yield value 4-6 Mg ha^-1^. The average yields were 6.8 and 6.1 Mg ha^-1^ in 2016 and 2017, respectively (**Figures 2A, B**). Most farmers preferred a planting density of 4.5-5 plants m^-2^, with 48.0% and 40.5% of the surveyed field maintaining this planting density in 2016 and 2017, respectively. The average planting density for 2016 and 2017 was 4.7 and 4.8 plants m^-2^ (**Figures 2C, D**). In addition, most of the surveyed farmers practiced rotary tillage (**Figures 2E, F**). Annual N fertilizer usage ranged from 200-300 kg ha^-1^ in 2016 and 2017, with average of 240.9 and 251.9 kg ha^-1^, respectively (**Figures 2G, H**).

### Grain yield and yield components under field experiments

Our DC’s enhanced field management increased grain yield (**Table 1**). Notably, yields under DC increased by 10.7% and 46.8% in 2018 and 2019, respectively, compared to the CC. We found no statistical significance in 1000-KW between DC and CC. We recorded significantly higher effective ear number under DC than that under CC, while the grain number per ear decreased. The effective ear is a critical determinant of maize yield under DC.

**Table 1:** Grain yield and grain yield components of CC and DC.

Year	Treatment	Effective ear (×10^3^ ear·ha^-1^)	Grain number per ear	1000-GW (g)	Yield (Mg·ha^-1^)
2018	CC	51.69b	626.40a	251.60a	8.15b
	DC	63.92a	559.77b	252.15a	9.02a
2019	CC	46.02b	550.79a	281.83a	7.14b
	DC	65.87a	534.86a	297.39a	10.48a

Values are the average of three replicates. DC, Dense cultivation; CC, Common cultivation; 1000-GW, 1000 grains weight. Statistical analysis was carried out using the one-way ANOVA test in 2018 and 2019, respectively. Different letters denote significant differences (P < 0.05).

### LAI of different layers in the canopy and total LAI

**Figure 3:**
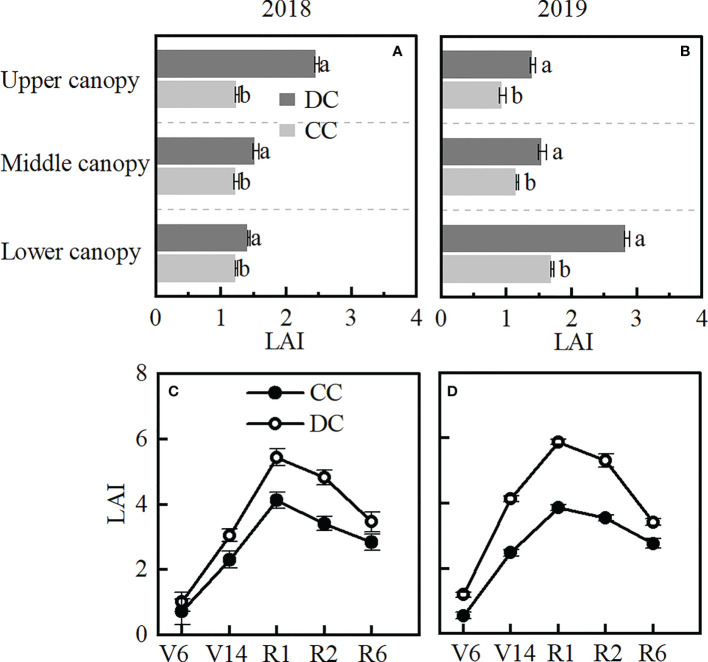
LAI of different layers of canopy, and total LAI in different stages. **(A, B)**: LAI of the upper, middle, and lower canopy at the R1 stage in 2018 and 2019. **(C, D)**: total LAI of different stages in 2018 and 2019. Upper canopy: above the three-ear leaves. Middle canopy: three-ear leaves. Lower canopy: below the three-ear leaves. DC, Dense cultivation. CC, Common cultivation. Different letters denote significant differences (*P* < 0.05), error bars show standard error of mean.

All LAI across different canopies under DC were higher than those recorded under CC across the two years (**Figures 3A, B**). The average two-year LAI in the upper (0.8), middle (0.3) and lower canopies (0.7) under DC significantly higher than that in CC. Similarly, the total LAI recorded in DC was significantly higher than CC at all the studied growth stages (V6, V14, R1, R2 and R6) (**Figures 3C, D**).

### Morphology of maize plants, LA and LOV

**Figure 4:**
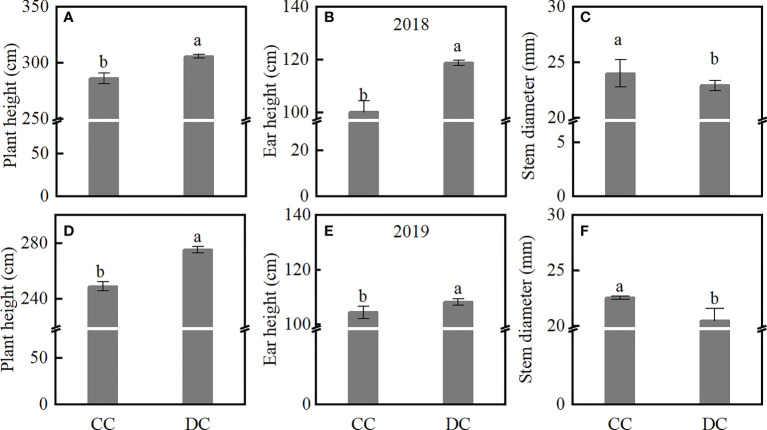
The morphology of the maize plants at the R1 stage. **(A–C)**: plant height, ear height, and stem diameter of CC and DC in 2018. **(D–F)**: plant height, ear height, and stem diameter of CC and DC in 2019. DC, Dense cultivation. CC, Common cultivation. Different letters denote significant differences (*P* < 0.05), error bars show standard error of mean.

The average plant height and ear height under DC was 8.5% and 11.1% higher than those under CC, across 2018 and 2019, respectively. However, stem diameter was lower under DC compared to CC across both years (**Figures 4A–F**). Next, we determined the LA and LOV across different canopies, and found that the LA of DC decreased under upper and middle canopy, while LOV increased in 2018 and 2019, compared to CC (**Table 2**). DC had reduced stem diameter and LA and increased plant height, ear height, and high LOV.

**Table 2:** Leaf angle and leaf orientation value of different canopies at the R1 stage.

Year	Treatment	Upper canopy		Middle canopy		Lower canopy	
		LA (°)	LOV	LA (°)	LOV	LA (°)	LOV
2018	CC	26.22a	55.31b	29.44a	51.93b	32.22a	46.57a
	DC	23.26b	57.76a	26.11b	53.17a	32.93a	48.40a
2019	CC	25.67a	52.77b	28.11a	45.60b	29.55a	42.84b
	DC	21.70b	70.64a	26.90b	60.80a	29.31a	56.06a

Values are the average of three replicates. DC, Dense cultivation. CC, Common cultivation. LA, leaf angle (the angle between leaf and stem). LOV, leaf orientation value. Upper canopy, above the three-ear leaves. Middle canopy, three-ear leaves. Lower canopy, below the three-ear leaves. Statistical analysis was carried out using the one-way ANOVA test in 2018 and 2019, respectively. Different letters denote significant differences (P < 0.05).

### Light distribution, transmittance, and interception rate

**Figure 5:**
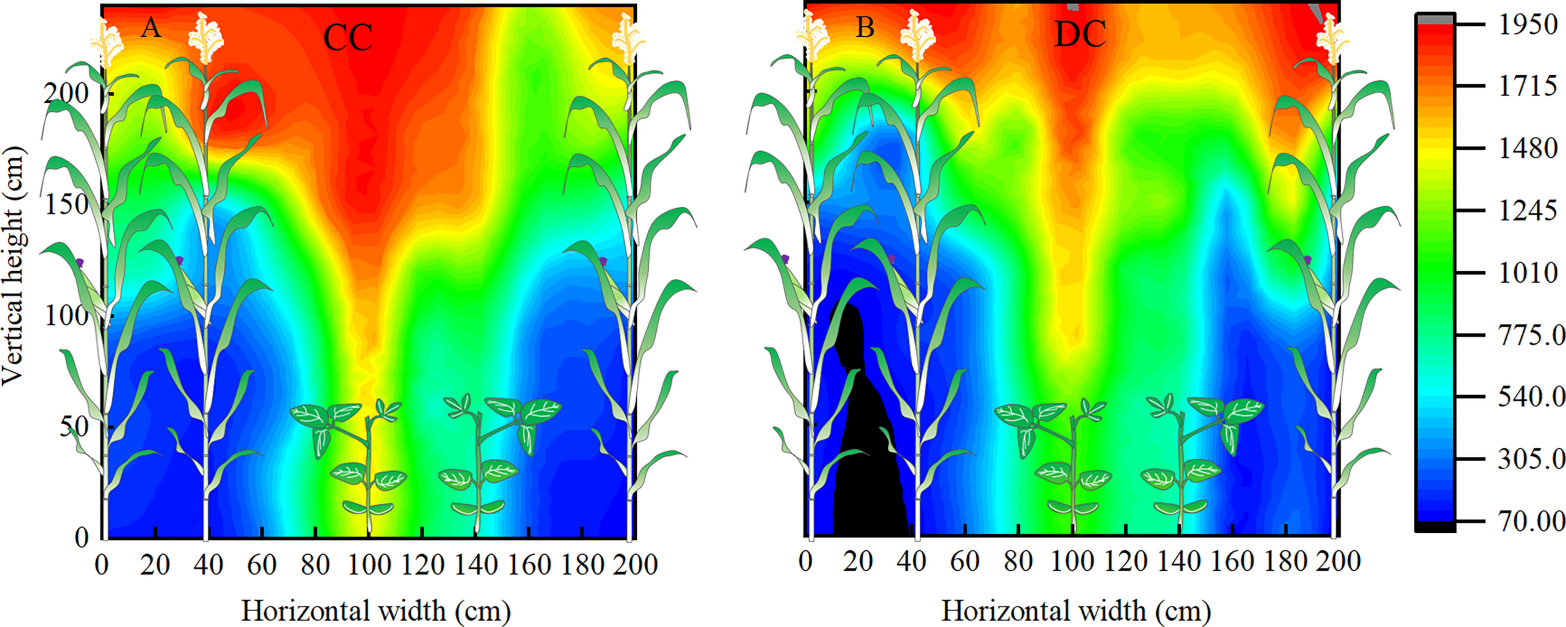
Light distribution in maize canopy. **(A, B)**: the light distribution of maize canopy in CC and DC at the R1 stage, respectively. DC, Dense cultivation. CC, Common cultivation. Plot values are photosynthetically active radiation (PAR, μmol m^-2^ s^-1^).

Results from light distribution tests revealed lower PAR in DC than CC, within 0-175 cm vertical and 0-40 cm horizontal area of the canopy, respectively. Particularly, PAR within vertical 75 cm was lower under DC, compared to CC (**Figures 5A, B**). Compared to CC, we noticed significantly lower transmittance in lower and middle canopies of DC, but there was no statistical difference between DC and CC with regards to transmittance and light interception in the upper canopy (**Table 3**). Moreover, it is worthy to notice that light interception rate of middle canopy in DC was significantly higher than CC.

**Table 3:** Transmittance and light interception rate in different canopy.

Treatment	Transmittance (%)			Light interception rate (%)		
	Upper canopy	Middle canopy	Lower canopy	Upper canopy	Middle canopy	Lower canopy
CC	91.37a	40.84a	23.31a	8.63a	50.52b	17.53a
DC	93.34a	27.83b	18.27b	6.66a	65.52a	9.56b

Values are the average of three replicates. DC, Dense cultivation. CC, Common cultivation. Upper canopy: above the three-ear leaves. Middle canopy: three-ear leaves. Lower canopy: below the three-ear leaves. Different letters denote significant differences (P < 0.05).

### Activities of PEP carboxylase and Rubisco

**Figure 6:**
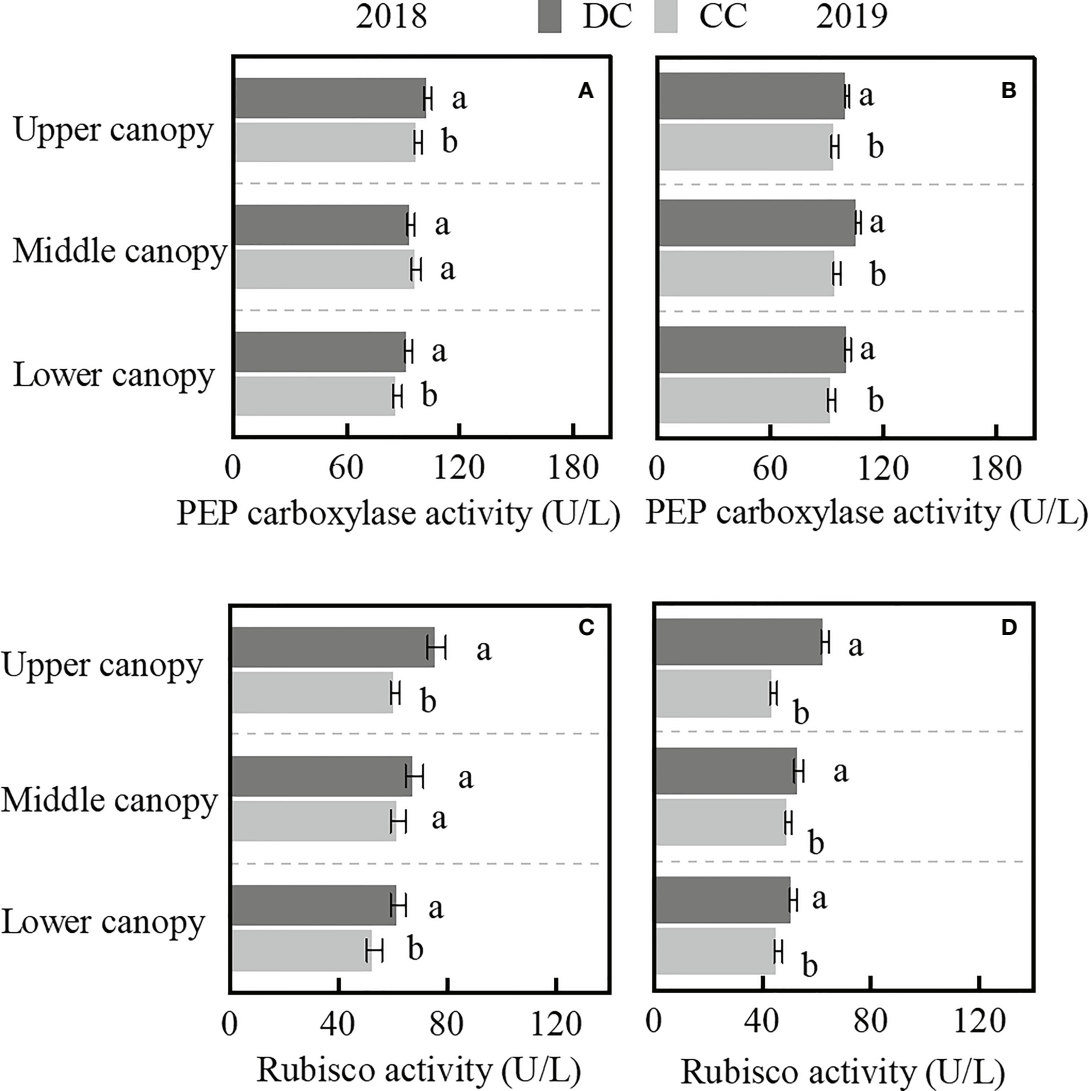
Activities of PEP carboxylase and Rubisco in the different canopy leaves at the R1 stage. **(A, B)**: PEP carboxylase activity of upper, middle, and lower canopy leaves in 2018 and 2019. **(C, D)**: Rubisco activity of differences canopy leaves in 2018 and 2019. DC, Dense cultivation. CC, Common cultivation. Different letters denote significant differences (*P* < 0.05), error bars show standard error of the mean.

In comparison to CC, PEP carboxylase activity was significantly higher in the upper and lower canopies of DC. On average, the activity was 6.1% and 7.8% higher in 2018 and 2019 in DC as compared to CC, respectively (**Figures 6A, B**). Similarly, the Rubisco activities in both upper and lower canopy leaves were higher in DC than in CC (**Figures 6C, D**). DC field management not only improved PEP carboxylase activities in the upper leaves but also the Rubisco activities of upper and lower canopy leaves.

### Pn and photosynthetic productivity

In the upper canopy, Pn was significantly greater in DC compared to CC (**Table 4**). However, no significant differences were observed between DC and CC with regards to Pn in the middle and lower canopies. DC recorded higher photosynthetic productivity in the upper, middle, lower, and total canopies were higher than CC in 2018 and 2019. The DC had a higher photosynthetic productivity across all canopies.

**Table 4:** Net photosynthetic rate and photosynthetic productivity in different canopy.

Year	Treatment	Pn (μmol CO_2_·m^-2^·s^-1^)			Photosynthetic productivity (Pn×LAI) (mg CO_2_ m^-2^ s^-1^)			
		Upper canopy	Middle canopy	Lower canopy	Upper canopy	Middle canopy	Lower canopy	Total
2018	CC	24.35b	19.45a	16.54a	21.67b	24.12b	19.85b	66.97b
	DC	26.32a	18.91a	17.64a	47.90a	29.88a	24.69a	100.59a
2019	CC	27.24b	27.12a	20.59a	20.31b	31.71b	39.25b	91.27b
	DC	31.62a	31.30a	21.76a	44.31a	53.75a	58.73a	156.79a

Values are the average of three replicates. DC, Dense cultivation. CC, Common cultivation. Upper canopy: above the three-ear leaves. Middle canopy: three-ear leaves. Lower canopy: below the three-ear leaves. Different letters denote significant differences (P < 0.05). Pn, net photosynthetic rate. LAI, leaf area index.

### Correlation analysis

**Figure 7:**
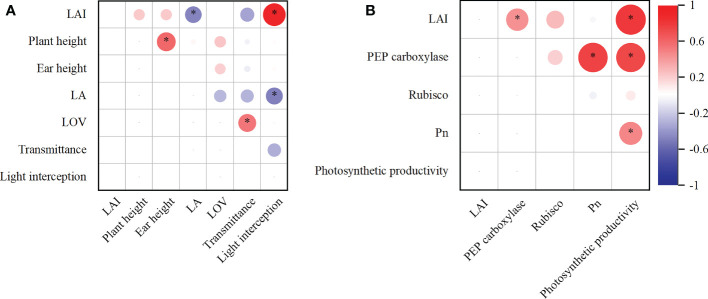
Correlation analyzed with light interception **(A)** and photosynthetic productivity **(B)**.

Results from correlation analyses are shown in **Figures 7A, B**. Summarily, light interception was significantly positively correlated (*P* < 0.05) with LAI, which showed a negatively correlated (*P* < 0.05) with LA. In addition, the Pn correlated significantly positively (*P* < 0.05) with PEP carboxylase activity. Similarly, a significant positive correlation (*P* < 0.05) was observed between photosynthetic productivity with Pn and LAI (**Figure 7B**).

## Discussion

### Farmer yield potential still has space for further improvement

The outcome of the survey showed that most farmer yields reached 4-6 Mg ha^-1^, with only four farmers achieving 10-12 Mg ha^-1^. This suggests that yield more than 10-12 Mg ha^-1^ is theoretically feasible (**Figures 2A, B**). Subsequently, we investigated the effect of field management and found that most of the farmers maintain planting density of 4.5-5 plant m^-2^. Moreover, rotary tillage was the local primary tillage modality, while the annual N fertilizer usage range from 200-300 kg ha^-1^ (average 246.4 kg ha^-1^). Numerous studies have shown that effective field management improves yield. Particularly, a high population density has excellent effect in maize by increasing radiation utilization efficiency and significantly improving grain yield potential (Liu et al., 2017; Gonzalez et al., 2018). Plough tillage increased grain yield, due to the deeper tillage depth reduced nutrition loss by surface runoff (Du et al., 2019). In Southwestern China, annual N fertilizer application in intercropping maize was found to be about 200-240 kg N ha^-1^ (Wang et al., 2017; Yang et al., 2017; Raza et al., 2019a). Accordingly, we designed DC comprising higher population density (6.75 plants m^-2^), plough tillage, and rational use of N fertilizer (270 kg N ha^-1^). This system resulted in an average yield increase of 28.8% compared to the CC. Although, it is common that high density and increased fertilization result in higher yield, but our study is more systemic as it is based on the results from an extensive survey that makes our DC more authentic and practical.

Solar radiation is vital for photosynthesis, while radiation intensity has a key role in determining the maize planting density in the local area (Zhang et al., 2006). Previous studies have suggested that CC in Southwestern China usually adopt the low density (4.8 plants m^-2^) system due to abundance of rainfall and low solar radiation (Ming et al., 2017). Other evidences have also shown that excessive rainfall is unfavorable to increase planting density, while high humidity is not conducive for seeding formation and also leads to vigorous growth as well as lodging (Ming et al., 2017). However, the results in present study clearly indicated that adjusting the field management significantly improves farm yields, and does not cause vigorous growth and lodging. In addition, DC yield was lower than the record for maximum yield from survey data. Four farmers have achieved highest yield; the most probable reason for this difference was different planting region. Another possible reason is application of farmyard manure.

To date, the yield potential of relay intercropped maize under low solar radiation area remains unclear. Some scholars applied model simulations to obtain maximum yield potential in Northwest China (where solar radiation is abundant), as evidenced by 12.0 Mg ha^-1^, farmer yields was 51% lower than maximum yields potential (Gou et al., 2017). In the present study, we obtained an average yield 9.8 Mg ha^-1^ under DC system, which was 18.3% lower than the potential yield in Northwest China. Remarkably, Southwest China has lower solar radiation compared to suitable global areas for crop planting, and annual precipitation is 1009.4 mm, which is 3.9-fold in Northwest China (Gou et al., 2017; Liu et al., 2017). Low solar radiation and high precipitation led to a decrease in yield potential under DC. Additionally, we obtained more yield potential under the DC system than what has been reported in many previous studies on maize intercropping in Southwestern China (Wang et al., 2017; Chen et al., 2019; Feng et al., 2019; Raza et al., 2019a; Raza et al., 2019b; Feng et al., 2020). Although we did not achieve the maximum yield potential of maize intercropping, the DC system mediated a marked increase in yield as compared to the CC and what has been reported in previous studies. Based on these findings, it is evident that increasing planting density and fertilization as well as adopting plough tillage can improve yield potential in Southwest China.

### Canopy structure under DC improved the light interception

Capture of a crop’s light energy is determined by canopy light interception (Liu et al., 2021b). Analysis of canopy structure is an effective way to evaluate light interception ability (Subedi and Ma, 2005). Light interception and LA are closely related, with optimal LA observed to improve light interception of the rice canopy. (Hammer et al., 2009; Sher et al., 2018; Xu et al., 2018). Additionally, higher LAI and LOV mean higher light interception, which is also the case for plant height (Ma et al., 2014; Hu et al., 2016; Senapati et al., 2019). Results of the present study indicated that the DC system resulted in higher plant height than CC, as well as higher LAI and LOV across all the canopies. On the other hand, transmittance of middle and lower canopy declined in DC while light interception rate increased (**Tables 2** and **Table 3**). To find out whether canopy structure plays a role increasing light interception, we further correlated canopy structure and light interception. Results showed that light interception had a significant negative correlation with LA, but a significant positive correlation with LAI (**Figure 7**). These results indicate that both LA and LAI play a key role in determining light interception in the canopy. The low value of LA in the upper and middle canopies, higher value of LAI in the upper, middle, and lower canopies ensured high light interception in DC. Interestingly, why does decrease of LA under DC? Previous studies have shown that LA increased (leaf inclination angles decrease) with leaf weight and area (Hernández, 2010). Modification in leaf orientation suggest shade avoidance reactions by a reduction in the red:far-red ratio of light in the canopy (Maddonni et al., 2001). As a result, a decrease in leaf weight and area per plant, as well as shade avoidance behaviors, may be major factors contributing to decreased LA in DC.

### Canopy structure of DC improved photosynthetic productivity

The photosynthetic productivity under the DC system improved due to the Pn of upper canopy and an increase in the LAI of all canopies (**Table 4** and **Figure 3**). Similarly, activities of two major enzymes, namely PEP carboxylase and Rubisco (Paulus et al., 2013; Atkinson et al., 2020), were high in the upper and lower canopies (**Figure 6**). Additionally, photosynthetic productivity exhibited a significant positive correlation with Pn and LAI, with Pn also showing a significant positive correlation with PEP carboxylase activity (**Figure 7**). The increase in PEP carboxylase activity in upper and lower canopies, coupled with Pn in the upper canopy, as well as elevated LAI across all canopies, generated a corresponding increase in photosynthetic productivity under DC.

The several layers in a maize canopy each serve a distinct functions. For instance, leaves around and above the ear commonly provide energy for grain development. Previous studies have shown that enhanced light interception in the middle canopy (100-150 cm) positively affects grain yield (Liu et al., 2011). In the present study, photosynthetic productivity increased under DC in the middle canopy, suggesting a possibility for increased yield. Additionally, we found that leaves at a height of 0-100 cm had improved light interception in wide-narrow row planting patterns in maize (**Figure 5**). These leaves provide photosynthates that aid in root development and growth (Liu et al., 2011), which subsequently have far-reaching implications for grain yield improvement. Under DC, lower canopy leaves (0-100 cm) exhibited higher Pn and photosynthetic productivity, which consequently enhanced grain yield.

Notably, the previous studies suggested that increase of intercropping grain yield was benefited by the complementarity effect (Gou et al., 2017; Li et al., 2020). The component crops in intercropping have a longer coexistence period than that in relay intercropping. The competition for nutrients is more important than aboveground competition for light in maize-soybean intercropping (Lv et al., 2014). In present relay intercropping of maize and soybean, coexistence period was relatively short (about 48 days), and with greater distance (60 cm) between strip of maize and soybean, which means the increased yield of DC was mainly due to an increase in light interception rate of maize middle canopy, rather than complementarity effect.

## Conclusions

In the current study, we surveyed 300 farmers and subsequently designed our experiment on the basis of survey data, to provide a realistic insight into the farmer yield and possible ways to increase the maize productivity in maize-soybean relay intercropping. Our findings indicate that increasing planting density and fertilization, as well as using a plough tillage system, can boost the yield potential of farmers’ existing farming practices. Moreover, our findings clearly indicate that optimizing canopy structure improved the light interception and photosynthetic productivity, which subsequently mediated a marked increase in grain yield. Improved LAI and compact LA effectively increases light interception and utilization. Taken together, this study presented a systemic experiment based on extensive survey of farmer fields to provide a practical solution for improving maize yields under the intercropping system, particularly in areas of low solar radiation. This study had some limitations, despite the substantial yield increases by improved field management observed in this study, it is still not enough to explore the potential yield completely. Future research, using new hybrids, irrigation systems, among others, are needed to validate the observed improvement in yield potential of crops under intercropping systems.

## Data availability statement

The original contributions presented in the study are included in the article/**Supplementary Material**. Further inquiries can be directed to the corresponding author.

## Author contributions

Writing-original draft: GC. Data curation: YR, BL, and HC. Writing, revision and methodology: AM and HG. Formal analysis: TY and JL. Project administration: XS and TP. Software: YW. Supervision: WL, WY. Resources: JD and FY. Funding acquisition: XW. All authors contributed to the article and approved the submitted version.

## Funding

This study was supported by the National Key Research and Development Program of China (2016YFD 0300109-3) and Science and Technology Program Project of Sichuan Province (2016NYZ0051).

## Conflict of interest

The authors declare that the research was conducted in the absence of any commercial or financial relationships that could be construed as a potential conflict of interest.

The reviewer [GA] declared a shared affiliation with one of the authors [AM] to the handling editor at the time of review.

## Publisher’s note

All claims expressed in this article are solely those of the authors and do not necessarily represent those of their affiliated organizations, or those of the publisher, the editors and the reviewers. Any product that may be evaluated in this article, or claim that may be made by its manufacturer, is not guaranteed or endorsed by the publisher.
