# Supplementary material for: Comparative analysis of farmer practices and high yield experiments: Farmers could get more maize yield from maize-soybean relay intercropping through high density cultivation of maize
Source: Front Plant Sci. 2022 Nov 15;13:1031024. doi: 10.3389/fpls.2022.1031024 (PMC9706207; doi:10.3389/fpls.2022.1031024)
Supplement: Supplementary file 1 [file Table_1.docx]

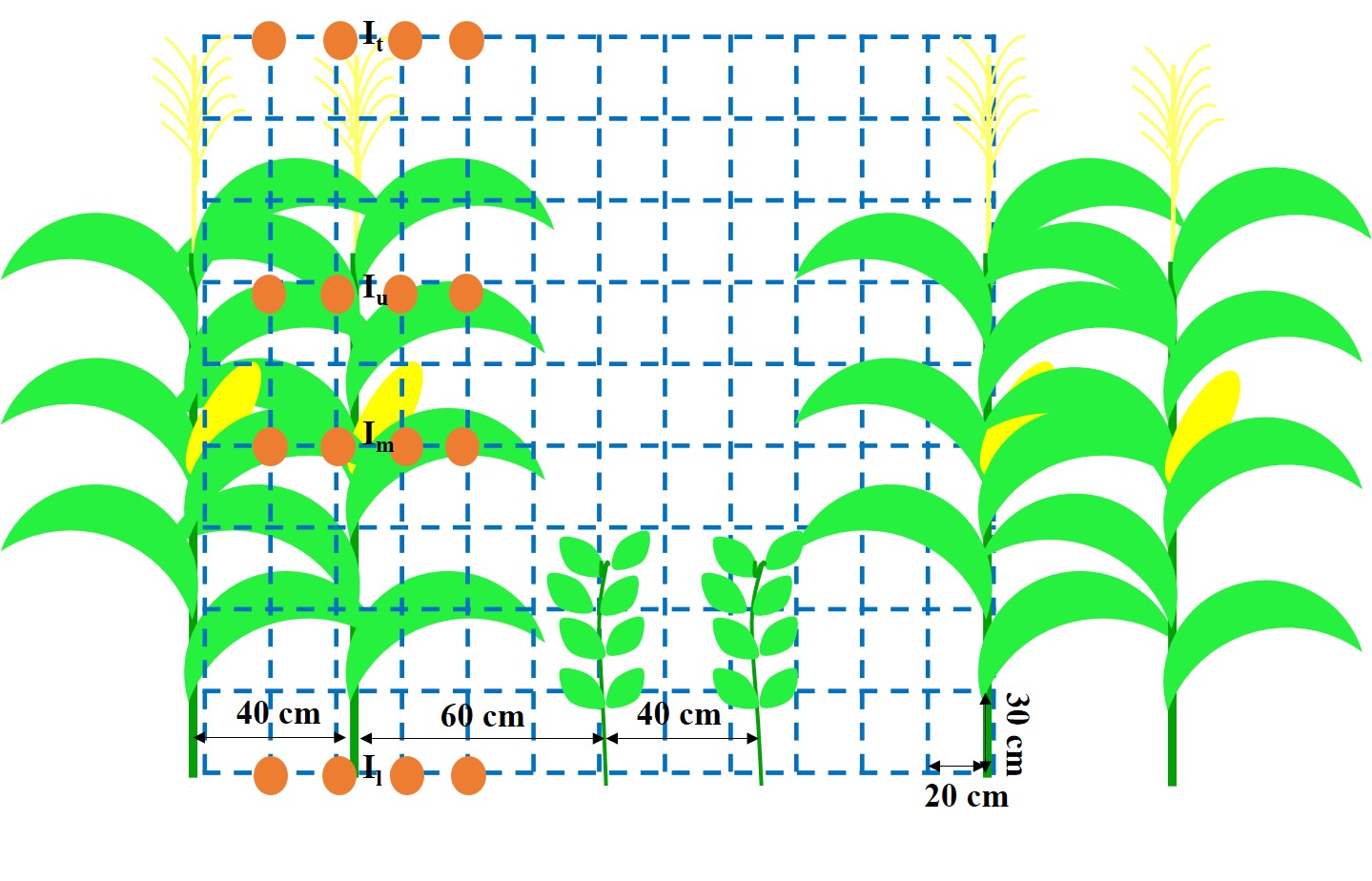


Figure S1 Schematic diagram of location of PAR measurement. The data in the horizontal and vertical directions were measured interval 20 cm and 30 cm, respectively. The four points (orange circle) PAR of each layer were used to calculate the means value of PAR in the top (I_t_), upper (I_u_), middle (I_m_), and lower canopies (I_l_), respectively.
